# Exploring convolutional neural networks for drug–drug interaction extraction

**DOI:** 10.1093/database/bax019

**Published:** 2017-05-25

**Authors:** Víctor Suárez-Paniagua, Isabel Segura-Bedmar, Paloma Martínez

**Affiliations:** Department of Computer Science, University Carlos III of Madrid Leganés 28911, Madrid, Spain

## Abstract

Drug–drug interaction (DDI), which is a specific type of adverse drug reaction, occurs when a drug influences the level or activity of another drug. Natural language processing techniques can provide health-care professionals with a novel way of reducing the time spent reviewing the literature for potential DDIs. The current state-of-the-art for the extraction of DDIs is based on feature-engineering algorithms (such as support vector machines), which usually require considerable time and effort. One possible alternative to these approaches includes deep learning. This technique aims to automatically learn the best feature representation from the input data for a given task. The purpose of this paper is to examine whether a convolutional neural network (CNN), which only uses word embeddings as input features, can be applied successfully to classify DDIs from biomedical texts. Proposed herein, is a CNN architecture with only one hidden layer, thus making the model more computationally efficient, and we perform detailed experiments in order to determine the best settings of the model. The goal is to determine the best parameter of this basic CNN that should be considered for future research. The experimental results show that the proposed approach is promising because it attained the second position in the 2013 rankings of the DDI extraction challenge. However, it obtained worse results than previous works using neural networks with more complex architectures.

## Introduction

There is currently a growing concern about adverse drug events (ADEs), which are a serious risk for patient safety (1) as well as a cause of rising health-care costs (2). Drug–drug interactions (DDIs), which are a type of ADE, are undesirable effects caused by the alteration of the effects of a drug due to recent or simultaneous use of one or more other drugs. Unfortunately, most DDIs are not detected during clinical trials. Although clinical trials are designed to ensure both safety and effectiveness of a new drug, it is not possible to test all of its possible combinations with other drugs (3).

The early detection of clinically important DDIs is a very challenging task for health-care professionals because of the overwhelming amount of related information that is currently available (4). Physicians have to spend a long time reviewing DDI databases as well as the pharmacovigilance literature in order to prevent harmful DDIs. The number of articles published in the biomedical domain increases by 10 000–20 000 articles weekly (http://www.nlm.nih.gov/pubs/factsheets/medline.html). Each year, 300 000 articles are published within only the pharmacology domain (5). Information extraction (IE) from both structured and unstructured data sources may significantly assist the pharmaceutical industry by enabling the identification and extraction of relevant information as well as providing a novel way of reducing the time spent by health-care professionals to review the literature. Most of the previous research on the extraction of DDIs from biomedical texts has focused on supervised machine-learning algorithms and extensive feature sets, which are manually defined by text miners and domain experts. Deep learning methods are potential alternatives to classical supervised machine-learning algorithms because they are able to automatically learn the most appropriate features for a given task. In particular, the hypothesis is that a convolutional neural network (CNN) may be an effective method to learn the best feature set to classify DDIs without the need for manual and extensive feature engineering. Although previous works have already incorporated the use of CNNs for DDI extraction (6, 7), none of them reported a detailed study of the influence of the CNN hyper-parameters on the performance. Similarly, to the best of our knowledge, there has been no focus on evaluating the CNN-based approach for each DDI type on the different types of texts, such as scientific articles (e.g. MedLine abstracts) or drug package inserts (e.g. text fragments contained in the DrugBank database).

### Related work

In recent years, several natural language processing (NLP) challenges have been organized to promote the development of NLP techniques applied to the biomedical domain, especially pertaining to the area of pharmacovigilance. In particular, the DDIExtraction shared tasks (8, 9) were developed with two objectives: advancing the state-of-the-art of text-mining techniques applied to the pharmacovigilance domain, and providing a common framework for the evaluation of the participating systems and other researchers who may be interested in the extraction of DDIs from biomedical texts. In 2011, the first edition addressed only the detection of drug DDIs, but the second edition also included their classification. Each DDI is classified according to one of the following types of DDIs: *mechanism* (when the DDI is described by their pharmacokinetic (PK) mechanism), *effect* (for DDIs describing an effect or a pharmacodynamic (PD) mechanism), *advice* (when a sentence provides a recommendation or advice about a DDI) and *int* (the DDI appears in the text without providing any additional information). Most of the participating systems as well as the systems that were subsequently developed, have been based on support vector machines (SVMs) and on both linear and non-linear kernels, and obtained state-of-the-art performance F1-scores of 77.5% for detection and 67% for classification (10). All of them are characterized by the use of large and rich sets of linguistic features, which have to be defined by domain experts and text miners, and which require considerable time and effort. The top system in the DDIExtraction Shared Task 2013 was developed by the Fondazione Bruno Kessler team (FBK-irst) (11). The system consisted of two phases: first, the DDIs were detected, after which they were classified according to the four types presented above. In the DDI-detection phase, filtering techniques based on the scope of negation cues and the semantic roles of the entities involved were proposed to rule out possible negative instances. In particular, a binary SVM classifier was trained using contextual and shallow linguistic features to determine these negative instances, which were not considered in the classification phase. Once these negative instances were discarded from the test dataset, a hybrid kernel [combining a feature-based kernel, the shallow linguistic kernel (SL) (12) and the path-enclosed tree (PET) kernel (13)] was used to train a relation extraction (RE) classifier. For the classification of the DDIs, four separate SVM models were trained for each DDI type (using ONE-vs-ALL). The experiments showed that the filtering techniques improved both the precision and recall compared to the case when only the hybrid kernel was applied. During the classification, the system obtained an F1-score of 65.1, 70.5 and 38.3% over the whole database, DrugBank and MedLine, respectively. This system was unable to classify the DDI relations in sentences such as: *Reduction of PTH by****cinacalcet****is associated with a decrease in****darbepoetin****requirement* [False Negative (FN)] and *There are no clinical data on the use of****MIVACRON****with other****non-depolarizing neuromuscular blockingagents*** [False Positive (FP)]. The above false negative may be due to this DDI is described by a complex syntactic structure. As mentioned before, one of the kernels used by the system is the PET kernel, which heavily relies on syntactic parsing. For the false FP, the main problem may be that the system failed to correctly identify the scope of negation in the sentence. Our work aims to overcome these problems by using a method that does not require the use of syntactic information. A full discussion on the main causes of errors in the DDIExtraction-2013 challenge task can be found in Ref. (14).

Afterwards, the work described in Ref. (10) overcame the FBK-irst’s top ranking system using a linear SVM classifier with a rich set of lexical and syntactic features, such as word features, word-pair features, dependency-parse features, parse-tree features and noun phrase-constrained coordination features to indicate whether the target drugs are coordinated in a noun phrase. To ensure generalization of these features, references to the target drug and the remaining drugs in the sentence are omitted. As part of the pre-processing, numbers and tokens contained in the sentences are replaced by a generic tag and normalized into lemmas, respectively. Every pair of drugs in a sentence is considered as a candidate DDI. Those candidates including the same drug name, e.g. (aspirin, aspirin), were directly removed. The system follows the two-phase approach (detection and classification) proposed by Chowdhury and Lavelli (11), but uses the ONE-vs-ONE strategy for the DDI-type classification because it increases the performance for the imbalanced dataset. They obtained an F1-score of 67% for the classification task.

The prominent use of deep learning in NLP and its good performance in this field makes it a promising technique for the task of RE. Matrix-vector recursive neural network (MV-RNN) (15), recurrent neural network (16) and convolutional neural network (CNN) (17) have been successfully applied to RE tasks.

The MV-RNN model was the first work in RE using a deep learning architecture which achieved state-of-the-art results on the SemEval-2010 Task 8 dataset (18). Following this approach, the work of Ebrahimi and Dou (19) demonstrates that using dependency parse instead of constituency parse in an RNN model improved the performance as well as the training time. They modified the RNN architecture in order to incorporate dependency graph nodes in which each dependency between entities has a unique common ancestor. In addition, they added some internal features from the built structure, such as the depth of the tree, distance between entities, context words, and the type of dependencies. They also evaluated their approach on the DDIExtraction 2013 dataset [the DDI corpus (20)], obtaining an F1-score of 68.64%. It should be noted that these authors did not carry out an in-depth study of the performance of their system for each type of DDI and for each of the subcorpora (DDI-DrugBank and DDI-MedLine) which comprise the DDI corpus. A more comprehensive study about MV-RNN for DDI extraction can be found in Ref. (21). This work concluded that MV-RNN achieved very low performance for biomedical texts because it uses the syntactic trees, which are generated by the Stanford Parser, as input structures. In general, these syntactic trees are incorrect because this parser has not been trained to parse biomedical sentences, which are usually very long sentences with complex structures (such as subordinate clauses, appositions and coordinate structures). The results obtained are different to those presented in Ref. (19) because these authors did not describe the setting for this method, such as the values of the hyper-parameters and the preprocessing phase, and did not clarify if their results were for the task of DDI detection or for DDI classification.

CNN is a robust deep-learning architecture which has exhibited good performance in many NLP tasks such as sentence classification (22), semantic clustering (23) and sentiment analysis (24). One of its main advantages is that it does not require the definition of hand-crafted features; instead, it is able to automatically learn the most suitable features for the task. This model combines the word embeddings of an instance (i.e. a sentence or a phrase containing a candidate relation between two entities) using filters in order to construct a vector which represents this instance. Finally, a softmax layer assigns a class label to each vector. Zeng et al. (17) developed the first work that used CNN for RE using the SemEval-2010 Task 8 dataset (18). This work concatenated the word embeddings with a novel position embedding which represents the relative distances of each word to the two entities in the instance relation in a embedding vector. In addition, they added a non-linear layer after the CNN architecture to learn more complex features attaining an F1-score of 69.7%. They obtained an improvement of 13% by adding external lexical features such as the word embeddings of the entities, their WordNet hypernym and the word embeddings of the context tokens.

Following these works, Liu et al. (9^6^) demonstrated that the use of CNN can outperform the rest of machine-learning techniques using pre-trained word embeddings and position embeddings trained with a large amount of documents from the biomedical domain. Currently, this work is the state-of-the-art system in the DDI classification task, with an F1-score of 69.75%. They also obtained a good performance for each DDI type: 70.24% for *mechanism*, 69.33% for *effect*, 77.75% for *advice* and 46.38% for *int*. Recently, the syntax CNN proposed by Zhao et al. (7) included a new syntax word embedding and a part-of-speech feature as an embedding, both of which are pre-trained with an autoencoder. Moreover, they also added some traditional features (such as the drug names, their surrounding words, the dependency types and the biomedical semantic types) to the softmax layer, and they used two-step classification (detection and classification). However, this system, which has an F1 of 68.6%, did not improve on the results reported in Ref. (6).

Table 1 summarizes the state-of-the-art systems for DDIExtraction Task. From the review of the related work, although some works have already applied the CNN model to the classification of DDIs, none of them involved a detailed study of the effect of its hyper-parameters by fine-tuning the performance of the model. In addition, unlike previous works, our system does not employ any external feature for the classification of DDIs. We also studied in detail the results of the CNN model for each type of DDI and on each dataset of the DDI corpus, i.e. DDI-DrugBank and DDI-MedLine. This study is required because these datasets involve very different types of texts (i.e. scientific texts versus drug package inserts).

**Table 1:** State-of-the-art systems results using the whole DDI Corpus for the classification task

Systems	Precision	Recall	F1-score
Liu *et al.* [6]	75.72%	64.66%	69.75%
Ebrahimi and Dou [19]	75.31%	66.19%	68.64%
Zhao *et al.* [7]	72.5%	65.1%	68.6%
Kim *et al.* [10]	Unknown	Unknown	67%
Chowdhury and Lavelli [11]	64.6%	65.6%	65.1%

As mentioned above, some previous works based on deep learning used syntactic information (7, 19). Biomedical sentences are usually long sentences and contain complex syntactic structures, which current parsers are not able to correctly analyze. In addition, syntactic parsing is a very time-consuming task, and hence may be infeasible in real scenarios. For this reason, in this work we explore an approach that does not require syntactic information. Our approach is similar to (6), however we perform a more detailed study. These authors initialized their CNN model with a pre-trained word embedding model from Medline (which is not publicly available) and only performed experiments with the default filter-size recommended by Kim (22). We explore not only several word embeddings models, but also a random initialization of the word vectors. In addition, one of our hypothesis is that because biomedical sentences describing DDIs are usually very long and their interacting drugs are often far from each other (the average distance between entities for all the instances in the train set is 14.6), we should try different window sizes to adapt this parameter to biomedical sentences. Moreover, unlike to the work (6), which only provided results for each DDI type on the whole test set, we provide the performance of our system for each DDI type and for each dataset of the DDI corpus (DDI-DrugBank and DDI-MedLine).

## Materials and methods

### Dataset

The major contribution of the DDIExtraction challenge was to provide a benchmark corpus, the DDI corpus. The DDI corpus is a valuable annotated corpus which provides gold standard data for training and evaluating supervised machine-learning algorithms to extract DDIs from texts. The whole DDI corpus contains 233 selected abstracts about DDIs from MedLine (DDI-MedLine) as well as 792 other texts from the DrugBank database (DDI-DrugBank). The corpus was annotated manually with a total of 18 502 pharmacological substances and 5028 DDIs. The quality and consistency of the annotation process was guaranteed through the creation of annotation guidelines, and it was evaluated by measuring the inter-annotator agreement (IAA) between two annotators. It should be noted that IAA can be considered as an upper bound on the performance of the automatic systems for detection of DDIs. The agreement was very high for the DDI-DrugBank dataset (Kappa = 0.83), and it was moderate for the DDIs in DDI-MedLine (0.55–0.72). This is because MedLine abstracts have a much higher complexity than texts from the DrugBank database, which are usually expressed in simple sentences. A detailed description of the method used to collect and process documents can be found in Ref. (25). The corpus is distributed in XML documents following the unified format for PPI corpora proposed by Pyysalo et al. (26). A detailed description and analysis of the DDI corpus and its methodology are described in Ref. (20).

**Figure 1:**
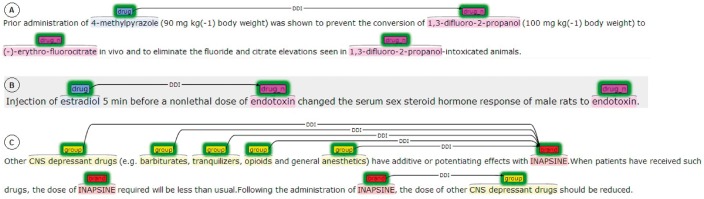
Some examples of sentences in the DDI corpus [14].

Figure 1 shows some examples [in brat format (http://brat.nlplab.org/)] of annotated texts in the DDI corpus. The first example (A) describes a *mechanism*-type DDI between a drug (4-methylpyrazole) that inhibits the metabolism of the substance (1,3-difluoro-2-propranol). The second example (B) describes the consequence of an *effect*-type DDI between estradiol and endotoxin in an experiment performed in animals. The first sentence of the last example (C) describes the consequence of the interaction (*effect* type) of a drug (Inapsine) when it is co-administered with five different groups of drugs. The third sentence in C shows a recommendation to avoid these DDIs (*advice* type). Table 2 shows the distribution of the DDI types in the DDI corpus.

**Table 2:** DDI types in the DDI corpus

DDI types	DDI-DrugBank	DDI-MedLine	Total
Advice	1035 (22%)	15 (4.6%)	1050 (20.9%)
Effect	1855 (39.4%)	214 (65.4%)	2069 (41.1%)
Int	272 (5.8%)	12 (3.7%)	284 (5.6%)
Mechanism	1539 (32.7%)	86 (26.3%)	1625 (32.3%)
Total	4701	327	5028

### CNN model

This approach is based on the CNN model proposed in Ref. (22), which was the first work to exploit a CNN for the task of sentence classification. This model was able to infer the class of each sentence, and returned good results without the need for external information. To this end, the model computes an output vector, which describes the whole sentence, and applies convolving filters to the input through several windows of different sizes. Finally, this vector is used in a classification layer to assign a class label. In this section, we present this model for the special case of sentences which describe DDIs. Instead of using Kim’s CNN implementation (https://github.com/yoonkim/CNN_sentence) based on Theano (a python library for mathematical computation (http://deeplearning.net/software/theano/), we adapt the implementation provided by Denny Britz (https://github.com/dennybritz/cnn-text-classification-tf) based on TensorFlow [an open-source library for machine learning (https://www.tensorflow.org/)]. TensorFlow has a graphic visualization of the model, and generates summaries of the parameters to keep track of their values, thus simplifying the study of the parameters.

**Figure 2:**
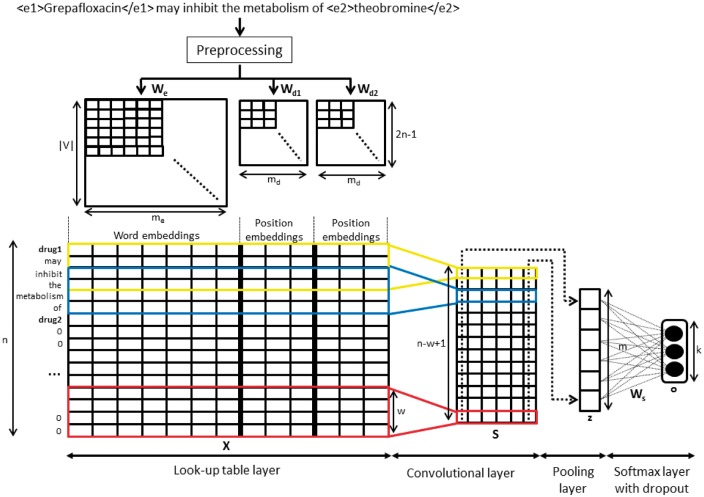
CNN model for DDIExtraction task.

Figure 2 shows the whole process from its input, which is a sentence with marked entities, until the output, which is the classification of the instance into one of the DDI types.

### Pre-processing phase

Each pair of drugs in a sentence represents a possible relation instance. Each of these instances is classified by the CNN model.

The DDI corpus contains a very small number of discontinuous drug mentions (only 47). An example of discontinuous mention is exemplified in the following noun phrase *ganglionic or peripheral adrenergic blocking drugs*, which contains two different drug mentions: *ganglionic adrenergic blocking drugs* and *peripheral adrenergic blocking drugs*, with the first one being a discontinuous entity. As this kind of mentions only produces a very small percentage (1.26%) of the total number of instances, we decided to remove them. The detection and classification of DDIs involving discontinuous drug mentions is a very challenging task, which will be tackled in future work.

First, following a similar approach as that described in Ref. (22), the sentences were tokenized and cleaned (converting all words to lower-case and separating special characters with white spaces by regular expressions.). Then, the two drug mentions of each instance were replaced by the labels ‘drug1’ and ‘drug2’ for the two interacting entities, and by ‘drug0’ for the remaining drug mentions. This method is known as entity blinding, and verifies the generalization of the model. For instance, the sentence: *Amprenavir significantly decreases clearance of rifabutin and 25-O-desacetylrifabutin* should be transformed to the following relation instances.

1. ‘drug1 significantly decreases clearance of drug2 and drug0’ for the relation (Amprenavir, rifabutin);
2. ‘drug1 significantly decreases clearance of drug0 and drug1’ for the relation (Amprenavir, 25-O-desacetylrifabutin);
3. ‘drug0 significantly decreases clearance of drug1 and drug2’ for the relation (rifabutin, 25-O-desacetylrifabutin).

### Word table layer

After the pre-processing phase, we created an input matrix suitable for the CNN architecture. The input matrix should represent all training instances for the CNN model; therefore, they should have the same length. We determined the maximum length of the sentence in all the instances (denoted by *n*), and then extended those sentences with lengths shorter than *n* by padding with an auxiliary token ‘0’.

Moreover, each word has to be represented by a vector. To do this, we considered two different options: (a) to randomly initialize a vector for each different word, or (b) to use a pre-trained word embedding model which allows us to replace each word by its word embedding vector obtained from this model: $W_{e}\in {\mathbb{R}}^{|V|\times m_{e}}$ where *V* is the vocabulary size and *m*_e_ is the word embedding dimension. Finally, we obtained a vector $x=[x_{1};x_{2};\ldots ;x_{n}]$ for each instance where each word of the sentence is represented by its corresponding word vector from the word embedding matrix. We denote *p*_1_ and *p*_2_ as the positions of the two interacting drugs mentioned in the sentence.

The following step involves calculating the relative position of each word to the two interacting drugs, $i-p_{1}$ and $i-p_{2}$, where *i* is the word position in the sentence. For example, the relative distances of the word *inhibit* in the sentence shown in Figure 2 to the two interacting drug mentions *Grepafloxacin* and *theobromine* are 2 and −4, respectively. In order to avoid negative values, we transformed the range (−n+1,n−1) to the range (1,2n−1). Then, we mapped these distances into a real value vector using two position embedding $W_{d1}\in {\mathbb{R}}^{(2n-1)\times m_{d}}$ and $W_{d2}\in {\mathbb{R}}^{(2n-1)\times m_{d}}$. Finally, we created an input matrix $X\in {\mathbb{R}}^{n\times (m_{e}+2m_{d})}$ which is represented by the concatenation of the word embeddings and the two position embeddings for each word in the instance.

One of the objectives of this work was to study the effect of the pre-trained word embeddings on the performance of CNNs. Thus, in addition to the CNN with a random initialization, we trained a CNN with different pre-trained word embedding models. First, we pre-trained different word embedding models using the toolkit word2vec (27) on the BioASQ 2016 dataset (28), which contains more than 12 million MedLine abstracts. We used both architectures of word2vec, skip-gram and continuous bag-of-words (CBOW), and applied the default parameters used in the C version of the word2vec toolkit (i.e. minimum word frequency 5, dimension of word embedding 300, sample threshold 10-5 and no hierarchical softmax). In addition, we used different values for the parameters context window (5, 8 and 10) and negative sampling (10 and 25). For a detailed description of these parameters, refer to (27). We also trained a word embedding model (with the default parameters of word2vec) on the XML text dump of the English 2016 version of Wikipedia (http://mattmahoney.net/dc/text8.zip).

### Convolutional layer

Once we obtained the input matrix, we applied a filter matrix $f=[f_{1};f_{2};\ldots ;f_{w}]\in {\mathbb{R}}^{w\times (m_{e}+2m_{d})}$ to a context window of size *w* in the convolutional layer to create higher level features. For each filter, we obtained a score sequence $s=[s_{1};s_{2};\ldots ;s_{n-w+1}]\in {\mathbb{R}}^{(n-w+1)\times 1}$ for the whole sentence as

$$s_{i}=g(\sum_{j=1}^{w}f_{j}x_{i+j-1}^{T}+b)$$

where *b* is a bias term and *g* is a non-linear function (such as tangent or sigmoid). Note that in Figure 2, we represent the total number of filters, denoted by *m*, with the same size *w* in a matrix $S\in {\mathbb{R}}^{(n-w+1)\times m}$. However, the same process can be applied to filters with different sizes by creating additional matrices that would be concatenated in the following layer. The filter size is an important parameter in the CNN model, and may influence its performance because it directly defines the size of the vector, which represents each instance. Moreover, window contexts have been traditionally exploited by most relation-classification systems. In particular, a window with a size of 3 is widely adopted (12).

### Pooling layer

Here, the goal is to extract the most relevant features of each filter using an aggregating function. We used the max function, which produces a single value in each filter as $z_{f}=max\{s\}=max\{s_{1};s_{2};\ldots ;s_{n-w+1}\}$. Thus, we created a vector $z=[z_{1},z_{2},\ldots ,z_{m}]$, whose dimension is the total number of filters *m* representing the relation instance. If there are filters with different sizes, their output values should be concatenated in this layer.

### Softmax layer

Prior to performing the classification, we performed a dropout to prevent overfitting. We obtained a reduced vector $z_{d}$, randomly setting the elements of **z** to zero with a probability *p* following a Bernoulli distribution. After that, we fed this vector into a fully connected softmax layer with weights $W_{s}\in {\mathbb{R}}^{m\times k}$to compute the output prediction values for the classification as

$$o=z_{d}W_{s}+d$$

where *d* is a bias term; in the dataset, we have *k* = 5 classes (*advice*, *effect*, *int*, *mechanism* and non-DDI). At test time, the vector **z** of a new instance is directly classified by the softmax layer without a dropout.

### Learning

For the training phase, we need to learn the CNN parameter set *θ* = ($W_{e},\;W_{d1},\;W_{d2},\;W_{s},\;F_{m}$), where $F_{m}$are all of the *m* filters **f**. For this purpose, we used the conditional probability of a relation *r* obtained by the softmax operation as

$$p(r|x,\theta )=\frac{\;\text{exp}(o_{r})}{\sum_{l=1}^{k}\;\text{exp}\;(o_{l})}$$

to minimize the cross entropy function for all instances ($x_{i}$,*y_i_*) in the training set *T* as follows.

$$J(\theta )=\sum_{i=1}^{T}\;\text{log}\;p(y_{i}|x_{i},\theta )$$

In addition, we minimize the objective function by using stochastic gradient descent over shuffled mini-batches and the Adam update rule [29] to learn the parameters. Finally, we add *l*_2_-regularization for the weights of the softmax layer $W_{s}$to prevent over-fitting.

## Results and discussion

In this section, we summarize the evaluation results with our CNN model on the DDI corpus, and we provide a detailed analysis and discussion. The results were measured using the Precision (P), Recall (R) and F1-score (F1) for all of the categories in the classification. To investigate the effect of the different parameters, we followed an evaluation process to choose the best model, selecting the parameters separately in a validation set to obtain the best values. Due to the fact that the DDI corpus is only split into training and test datasets, we randomly selected 2748 instances (candidate pairs) (10%) from the training dataset at the sentence level, forming our validation set, which was used for all our experiments to fine-tune the hyper-parameters of the architecture.

To validate each setting, we performed a statistical significance analysis between the models. For this purpose, we tested the significance with the $\chi^{2}$ and *P*-value statistics. Two models produce different levels of performance whether $\chi^{2}$ is > 3.84 and *P*-value is lower than 0.05.

First, we show the performance in a learning curve to find the optimal number of epochs for which the system achieves the best results with the stopping criteria. Second, a basic CNN was computed using predefined parameters to create a baseline system, after which we analyze its results. Third, the effects of the filter size and the selection of different word embeddings and position embeddings were observed. Finally, a CNN model using the best parameters found in the above steps was created. In addition, for all the experiments, we define the remainder of the parameters using the following values:

- Maximal length *n* = 128.
- Filters for each window size *m* = 200.
- Dropout rate p=50%.
- *l*_2_-regularization = 3.
- Mini-batch size = 50.
- Rectified Linear Unit (ReLU) as the non-linear function *g*.

The parameter *n* is the maximum length in the dataset after the pre-processing phase, *m* is the same as in Ref. (6) and the rest of the parameters are the same as in Ref. (22).

### Learning curve

**Figure 3:**
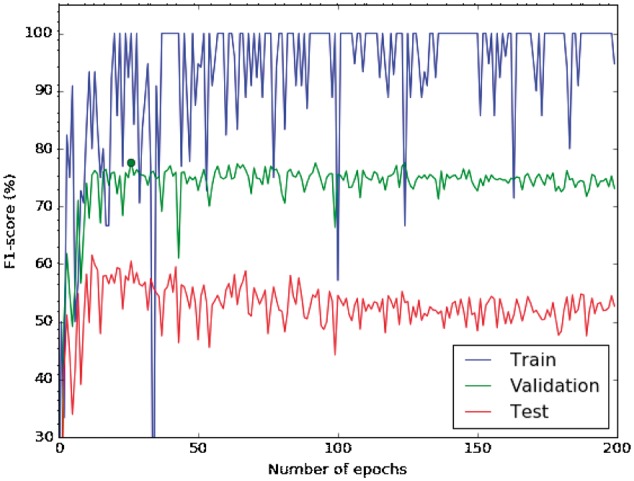
Learning curve of a CNN with random initialization. The blue line shows the training-curve variation along the number of epochs, the green represents the validation and the red one the testing curve.

Figure 3 shows the learning curve for our CNN from random initialization, i.e. instead of using pre-trained word embeddings as input features for our network, we generated random vectors of 300 dimensions using a uniform distribution in the range (−1,+1).

The curve shows the performance of each iteration of a learning step (epoch), and is measured in terms of F1 in the softmax layer. According to this learning curve, the best validation F1 is reached with 27 epochs (77.7%), which was identified as the optimum number of epochs (see the green point in Figure 3). Moreover, we observe that the training F1 is still around 100%, and the validation F1 does not improve by using more epochs. There is not a large gap between the training and validation F1, and therefore, the model does not appear to produce overfitting. Figure 3 also shows that the validation and test variation perform very similar, confirming that the choice of the parameters in the validation set is also valid for the test set. Finally, we used 25 epochs to train the network in the following experiments because after this point the model starts to decrease its performance. Moreover, it was the value chosen by Kim (22).

### Baseline performance

As previously mentioned, we trained our baseline CNN model from random initialization (i.e. without pre-trained word embeddings) of 300 dimensions, filter size (3, 4 and 5) and no position embeddings. The performance of this model for each of the DDI types is shown in Tables 4, 5 and 6. The model achieves an F1 of 61.98% on the DDI-DrugBank dataset, while its F1 on the DDI-MedLine dataset is lower (43.21%). This may be because the DDI-MedLine dataset (with 327 positive instances) is much smaller than the DDI-DrugBank dataset (with 4701 positive instances).

**Table 3:** Number of instances in each dataset of the DDI corpus after the pre-processing phase

DDI types	DDI-DrugBank	DDI-MedLine	Total
Advice	1028	14	1042
Effect	1815	214	2029
Int	272	12	284
Mechanism	1535	83	1618
Other	26 486	1892	28 373
Total	31 136	2215	33 351
Train	25 885	1778	27 663
Test	5251	437	5688

The class Other represents the non-interaction between pairs of drug mentions.

**Table 4:** Results obtained for CNN from random initialization on the whole DDI corpus

Classes	TP	FP	FN	Total	*P*	*R*	*F*_1_
Advice	131	43	90	221	75.29%	59.28%	66.33%
Effect	239	220	121	360	52.07%	66.39%	58.36%
Int	27	3	69	96	90%	28.12%	42.86%
Mechanism	176	84	122	298	67.69%	59.06%	63.08%
Overall	573	350	402	975	62.08%	58.77%	60.38%

**Table 5:** Results obtained for CNN from random initialization on the DDI-DrugBank dataset

Classes	TP	FP	FN	Total	*P*	*R*	*F*_1_
Advice	130	43	84	214	75.14%	60.75%	67.18%
Effect	212	190	86	298	52.74%	71.14%	60.57%
Int	27	2	67	94	93.1%	28.72%	43.9%
Mechanism	169	79	109	278	68.15%	60.79%	64.26%
Overall	538	314	346	884	63.15%	60.86%	61.98%

**Table 6:** Results obtained for CNN from random initialization on the DDI-MedLine dataset

Classes	TP	FP	FN	Total	*P*	*R*	*F*_1_
Advice	1	0	6	7	100%	14.29%	25%
Effect	27	30	35	62	47.37%	43.55%	45.38%
Int	0	1	2	2	0%	0%	0%
Mechanism	7	5	13	20	58.33%	35%	43.75%
Overall	35	36	56	91	49.3%	38.46%	43.21%

Next, we focus on the results obtained for each DDI type on the whole DDI corpus. The *advice* class is the type with the best F1. This can be explained because most of these interactions are typically described by very similar patterns such as *DRUG should not be used in combination with DRUG* or *Caution should be observed when DRUG is administered with DRUG*, which can be easily learned by the model because they are very common in the DDI corpus, especially in the DDI-DrugBank dataset. The *mechanism* type is the second one with the best performance (*F*_1_ = 63%), even though its number of instances is lower than the *effect* type (Table 4). While the systems which were involved in the DDIExtraction-2013 challenge agreed that the second easiest type was *effect* (14), this may have been because it was the second type with more examples in the DDI corpus; our model appears to obtain better performance for the *mechanism* type. As described in Herrero-Zazo *et al.* (20), one of the most common reasons for disagreement between the annotators of the DDI corpus is that a DDI is described by information related to both its *mechanism* and its *effect*, and thus the selection of the type is not obvious. For example, the sentence *Concomitant administration of TENTRAL and theophylline-containing drugs leads to increased theophylline levels and theophylline toxicity in some individuals* describes a change in the *mechanism* of the DDI (increased theophylline levels), as well as an *effect* (theophylline toxicity). In order to solve these cases, the annotators defined the following priority rule: first *mechanism*, second *effect* and third *advice*. While the systems developed so far have not been able to learn this rule, our CNN model appears to have acquired it correctly. Moreover, it should be noted that the sentences describing *mechanism* DDIs are characterized by the inclusion of PK parameters such as area under the curve (AUC) of blood concentration–time, clearance, maximum blood concentration (*C*_max_) and minimum blood concentration (*C*_min_). These kinds of parameters, which in general are expressed by a small vocabulary of technical words from the pharmacological domain, may be easily captured by the CNN model because the word vectors are fine-tuned for the training.

Finally, we observe that the *int* class is the most difficult type to classify. This may be because the proportion of instances of this type of DDI relationship (5.6%) in the DDI corpus is much smaller than those of the remainder of the types (41.1% for *effect*, 32.3% for *mechanism* and 20.9% for *advice*).

Tables 5 and 6 also show that the performance of each type is different depending of the dataset. Thus, while the above explanation can be extrapolated to the DDI-DrugBank dataset, the conclusions are completely different for the DDI-MedLine dataset. For example, the CNN model obtains lower results for the advice type (*F*_1_ = 25%) compared to the *effect* and *mechanism* types (with an *F*_1_ around 43–45%). This may be because the *advice* type is very scarce in the DDI-MedLine dataset. Likewise, our CNN model is unable to classify the *int* type, which is even scarcer than the *advice* type in this dataset.

### Filter-size selection

**Figure 4:**
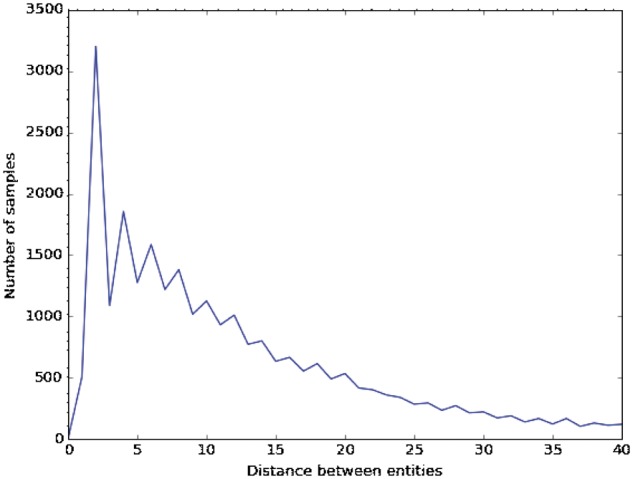
Distance between entities in sentences describing DDIs.

Figure 4 shows the distances between entities in the DDI corpus, which were obtained from >100 samples. We observe that the most common distances are 2, 4 and 6, with 3205, 1858 and 1586 samples, respectively. Because biomedical sentences describing DDIs are usually very long and their interacting drugs are often far from each other (the average distance between entities is 14.6), we used different window sizes to adapt this parameter to biomedical sentences.

Table 7 shows the results of our CNN baseline trained with different filter sizes. With the excepting of some cases (e.g. filter size = 2), most of the filter sizes provide very close results. In the case of a single filter size, 14 is the best one because it can capture long dependencies in a sentence with just one window. Although it seems logical to consider that larger filter sizes should give better performance, our experiments did not agree with this conclusion. Increasing the size appears to create incorrect filter weights, which cannot capture the most common cases. In fact, the best filter size was (2, 4 and 6), which may be because they are the most common distances between entities in the DDI corpus.

**Table 7:** Results for several filter sizes

Filter size	*P*	*R*	*F*_1_
2	56.89%	52.1%	54.39%
4	65.65%	52.92%	58.6%
6	**75.35**%	49.23%	59.55%
(2, 3, 4)	63.15%	57.13%	59.99%
(3, 4, 5)	62.08%	**58.77**%	60.38%
(2, 4, 6)	73.57%	52.82%	**61.49%**
(2, 3, 4, 5)	71.31%	52%	60.14%
14	71.23%	51.79%	59.98%
(13, 14, 15)	72.64%	49.03%	58.54%

Table 8 shows the significance tests for the experiments assessing the effect of filter-size parameter. In general, most of the comparisons are statistically significant, and especially those with the filter-size (2, 4 and 6) that achieves the best performance. Therefore, we conclude that the best performance is obtained using a filter-size of (2, 4 and 6). Thus, we can claim that the most frequent distances between entities are the best choice to be used as filter-size parameter.

**Table 8:** *χ*^2^ and P-value statistics between the different filter sizes

Filter size	4	6	(2, 3, 4)	(3, 4, 5)	(2, 4, 6)	(2, 3, 4, 5)	14	(13, 14, 15)
2	13.22*	50.68*	155.88*	1.20	25.77*	119.71*	44.52*	5.28*
	2.77e−04*	1.09e−12*	8.99e−36*	2.73e−01	3.84e−07*	7.32e−28*	2.52e−11*	2.16e−02*
4		20.01*	118.53*	21.92*	3.87*	97.79*	17.79*	0.14
		7.71e−06*	1.33e−27*	2.84e−06*	4.91e−02*	4.66e−23*	2.46e−05*	7.12e−01
6			44.14*	73.39*	7.92*	26.78*	0.08	22.25*
			3.06e−11*	1.06e−17*	4.89e−03*	2.28e−07*	7.73e−01	2.39e−06*
(2, 3, 4)				177.61*	69.08*	4.82*	33.78*	81.04*
				1.61e−40*	9.48e−17*	2.81e−02*	6.18e−09*	2.21e−19*
(3, 4, 5)					33.25*	146.78*	67.70*	11.33*
					8.12e−09*	8.75e−34*	1.91e−16*	7.65e−04*
(2, 4, 6)						51.97*	7.16*	5.06*
						5.63e−13*	7.45e−03*	2.45e−02*
(2, 3, 4, 5)							20.44*	67.10*
							6.16e−06*	2.58e−16*
14								31.01*
								2.57e−08*

Asterisk denotes results statistically significant.

### Effects of the embeddings

Table 9 shows the results for the different word embeddings as well as for several dimensions (5, 10) of position embeddings with a filter size (3, 4 and 5). As previously explained, position embedding enables us to represent the position of the candidate entities (which are involved in the DDI) as a vector. When the position embedding is not implemented in the model, we only use the word embedding as an input matrix.

**Table 9:** Performance with different word embedding and different position embedding size

Word embedding	Position embedding	*P*	*R*	*F*_1_
random	0	62.08%	58.77%	60.38%
	5	69.34%	55.9%	**61.9%**
	10	**70.76%**	54.36%	61.48%
Wiki_bow_8w_25n	0	60.89%	54.46%	57.5%
	5	59.2%	60.72%	59.95%
	10	70.64%	53.54%	60.91%
Bio_skip_8w_25n	0	62.39%	57.85%	60.03%
	5	67.8%	53.33%	59.7%
	10	66.92%	55.18%	60.48%
Bio_skip_10w_10n	0	70.66%	49.64%	58.31%
	5	61.84%	56.51%	59.06%
	10	68.77%	54.87%	61.04%
Bio_bow_8w_25n	0	64.09%	54.36%	58.82%
	5	69.43%	54.05%	60.78%
	10	67.27%	49.95%	57.33%
Bio_bow_5w_10n	0	58.25%	59.38%	58.81%
	5	60.18%	**61.23%**	60.7%
	10	65.21%	56.72%	60.67%

The prefix Wiki (Wikipedia corpus) or Bio (BioASQ dataset) refers to the corpus used to train the word embedding model. The label bow (CBOW) or skip (skip-gram) refers to the type of architecture used to build the model. The number preceding w and n indicates the size of the context window and the negative sampling, respectively.

In general, the implementation of position embeddings appears to realize a slight improvement in the results, providing the best scores when the dimension is 10. For example, for random initialization (i.e. the word vectors are randomly initialized and fine-tuned for the training), we observe that the inclusion of position embeddings achieves a slight increase in *F*_1_. In this case, the best *F*_1_ is achieved with a dimension of 5 for the position embedding. On the contrary, the CNN model which was trained using a word embedding model on Wikipedia (with a default setting in the C version of word2vec, which is represented as Wiki_bow_8w_25n in Table 9) appears to benefit from the implementation of position embeddings, achieving its best F1 (60.91%) with a dimension of 10 for the position embedding. Likewise, the CNN models, which were trained on the word embedding model from the BioASQ collection with skip-gram architecture (Bio_skip_8w_25n and Bio_skip_10w_10n), also provide better results when the dimension is 10. If the architectures are CBOW (Bio_bow_8w_25n and Bio_bow_5w_10n), the best F1 are obtained with dimension 5.

The results of training the CNN models on pre-trained word embeddings model from Wikipedia are slightly lower than those obtained with the model from random initialization. This may be because the word embedding learned from Wikipedia, which contain texts from a very wide variety of domains, may not be appropriate for the pharmacological domain. Neither of the word embedding models learned from the BioASQ collection (which focuses on the biomedical scientific domain) appear to provide better results than the CNN model initialized with random vectors. A possible reason for this may be that most texts in the DDI corpus are not scientific texts, but also fragments from health documents for patients, such as drug package inserts (which contain information about a given medication).

We also studied the effect of the word2vec parameters on the CNN performance. In Table 9, we observe that the two architectures (skip-gram and CBOW) provide very similar scores. However, it should be noted that the former has a very high computational complexity with a very long generation time compared to the latter, and CBOW therefore appears to be the best option to train our word embedding models. For more information about these architectures, refer to (27). For the CBOW architecture, the best *F*_1_ is 60.91% (window size 8 and negative sampling 25, trained on Wikipedia). When the same model trained on BioASQ, we obtained a very close *F*_1_ (60.78%).

The significance tests for the different word embeddings and position embeddings indicate that many of the comparisons are significant. In particular, our best model (whose word vectors were randomly initialized and the position embedding was set to 5) is statistically significant compared to the remainder models (Table 10).

**Table 10:** *χ*^2^ and *P*-value statistics between the different word embeddings and position embeddings

Word embedding and position embedding		random		Wiki_bow_8w_25n			Bio_skip_8w_25n			Bio_skip_10w_10n			Bio_bow_8w_25n			Bio_bow_5w_10n		
		5	10	0	5	10	0	5	10	0	5	10	0	5	10	0	5	10
random	0	78.41*	11.44*	0.74	11.41*	0.44	10.10*	0.14	20.98*	133.47*	0.26	0.02	127.06*	71.74*	10.71*	5.89*	132.98*	8.32*
		8.38e−19*	7.19e−04*	3.89e−01	7.31e−04*	5.08e−01	1.48e−03*	7.06e−01	4.64e−06*	7.13e−31*	6.08e−01	8.83e−01	1.80e−29*	2.46e−17*	1.06e−03*	1.52e−02*	9.12e−31*	3.93e−03*
	5		41.42*	43.25*	29.50*	78.75*	23.52*	46.94*	13.26*	23.89*	49.09*	40.16*	20.05*	1.48	20.31*	29.08*	23.86*	23.58*
			1.23e−10*	4.81e−11*	5.59e−08*	7.05e−19*	1.23e−06*	7.31e−12*	2.71e−04*	1.02e−06*	2.44e−12*	2.34e−10*	7.56e−06*	2.24e−01	6.60e−06*	6.94e−08*	1.04e−06*	1.20e−06*
	10			2.96	0.21	15.52*	0.26	8.78*	4.07*	92.25*	4.61*	6.76*	85.78*	40.50*	0.39	0.03	92.07*	0.03
				8.52e−02	6.45e−01	8.16e−05*	6.08e−01	3.05e−03*	4.37e−02*	7.64e−22*	3.18e−02*	9.32e−03*	2.01e−20*	1.97e−10*	5.33e−01	8.63e−01	8.36e−22*	8.56e−01
Wiki_bow_8w_25n	0				6.20*	3.09	7.15*	1.72	15.94*	124.17*	0.10	1.13	91.84*	46.65*	3.90*	1.57	100.12*	2.78
					1.28e−02*	7.90e−02	7.51e−03*	1.90e−01	6.54e−05*	7.74e−29*	7.54e−01	2.88e−01	9.41e−22*	8.47e−12*	4.83e−02*	2.10e−01	1.44e−23*	9.52e−02
	5					21.54*	0.00	9.43*	2.23	90.06*	7.56*	7.51*	66.94*	30.24*	0.01	0.43	76.09*	0.02
						3.46e−06*	1.00e+00	2.13e−03*	1.36e−01	2.31e−21*	5.96e−03*	6.13e−03*	2.80e−16*	3.81e−08*	9.41e−01	5.12e−01	2.71e−18*	8.76e−01
	10						17.78*	0.01	42.24*	144.40*	2.15	0.10	117.05*	76.26*	12.84*	8.05*	128.69*	12.34*
							2.48e−05*	9.34e−01	8.07e−11*	2.91e−33*	1.42e−01	7.55e−01	2.79e−27*	2.49e−18*	3.39e−04*	4.55e−03*	7.93e−30*	4.44e−04*
Bio_skip_8w_25n	0							14.78*	2.16	111.21*	11.72*	11.36*	61.25*	27.80*	0.00	0.49	69.84*	0.05
								1.21e−04*	1.41e−01	5.33e−26*	6.18e−04*	7.51e−04*	5.02e−15*	1.35e−07*	1.00e+00	4.82e−01	6.43e−17*	8.18e−01
	5								24.49*	139.36*	1.25	0.07	101.05*	63.22*	9.74*	5.81*	102.92*	8.84*
									7.47e−07*	3.67e−32*	2.63e−01	7.87e−01	8.98e−24*	1.85e−15*	1.81e−03*	1.59e−02*	3.49e−24*	2.95e−03*
	10									70.92*	26.30*	20.83*	52.75*	19.67*	1.06	3.63	55.44*	2.31
										3.72e−17*	2.93e−07*	5.03e−06*	3.79e−13*	9.19e−06*	3.03e−01	5.66e−02	9.63e−14*	1.28e−01
Bio_skip_10w_10n	0										131.16*	130.70*	0.12	9.66*	61.23*	90.72*	0.00	71.64*
											2.28e−30*	2.88e−30*	7.25e−01	1.88e−03*	5.09e−15*	1.66e−21*	1.00e+00	2.58e−17*
	5											0.69	93.74*	53.76*	5.60*	2.69	100.91*	4.50*
												4.07e−01	3.60e−22*	2.26e−13*	1.80e−02*	1.01e−01	9.61e−24*	3.39e−02*
	10												95.72*	55.37*	7.98*	4.37*	95.76*	7.06*
													1.33e−22*	9.96e−14*	4.72e−03*	3.66e−02*	1.30e−22*	7.90e−03*
Bio_bow_8w_25n	0													15.56*	89.27*	88.45*	0.11	82.26*
														8.01e−05*	3.44e−21*	5.22e−21*	7.43e−01	1.19e−19*
	5														36.01*	45.52*	12.88*	47.91*
															1.97e−09*	1.51e−11*	3.33e−04*	4.47e−12*
	10															0.71	90.31*	0.15
																4.00e−01	2.04e−21*	6.99e−01
Bio_bow_5w_10n	0																96.01*	0.20
																	1.15e−22*	6.56e−01
	5																	95.07*
																		1.84e−22*

Asterisk denotes results statistically significant.

### Optimal parameter performance

**Figure 5:**
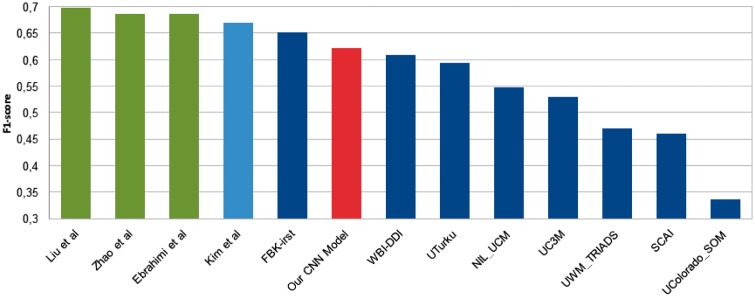
State-of-the-art F1-scores for DDI classification. The deep blue bars represents the participating system in DDIExtraction 2013. The light blue is the work of Kim *et al.* [10] and the green ones represent recent systems based on the deep learning techniques for DDI classification, which were subsequently presented. Our best model is represented by the red bar.

From the observation of the results on the validation set, it can be concluded that our best model has to be randomly initialized, with filter size (2, 4 and 6) and dimension of position embedding 5. Table 11 shows the results of this model for each type. The type with the best F1 is *advice* (71.25%), followed by *mechanism* (58.65%) and *effect* (58.65%). The worst type appears to be *int*, which has an F1 of only 41.22%. The possible causes for these results were previously discussed in this paper. The overall F1 is 62.23%. In Figure 5, we see that although our model does not achieve a new state-of-the-art F1 for DDI classification, it is very promising, and its results are comparable to those of previous systems.

**Table 11:** Results obtained for the best CNN model [random initialization, filter size (2, 4, 6) and position embedding of dimension 5] on the DDI corpus test set

Classes	TP	FP	FN	Total	*P*	*R*	*F*_1_
Advice	145	41	76	221	77.96%	65.61%	71.25%
Effect	183	81	177	360	69.32%	50.83%	58.65%
Int	27	8	69	96	77.14%	28.12%	41.22%
Mechanism	192	106	106	298	64.43%	64.43%	64.43%
Overall	547	236	428	975	69.86%	56.1%	62.23%

Finally, we performed a statistical significance analysis between the baseline system and the model with the optimal parameter values with the $\chi^{2}$and *P*-value statistics and obtained 5.7 and 0.017, respectively. These results suggest that the two models produce different levels of performance.

## Conclusions and future work

State-of-the-art methods for DDI extraction use classical supervised machine-learning algorithms (such as SVM) and intensive feature-engineering. We propose a CNN model to automatically learn features, which can be used to classify DDIs. The main contributions of this paper were as follows: (1) to make a detailed comparison of previous work for DDI extraction, (2) to provide an in-depth study of the influence of the CNN hyper-parameters on the results and (3) to evaluate the performance of a CNN model for different types of texts such as scientific articles and drug package leaflets as well as for the different type of DDIs.

Unlike some previous works based on deep learning (7, 19), our CNN model does not employ any external features in the classification layer. Their systems used external features such as the distance between the entities, the depth of the tree of the entities, the type of syntactic dependencies that links the entities or the contexts around the entities, among others. There is an extensive literature showing that these features can positively contribute to solve the relation extraction task. Consequently, if these external features were used, it would be difficult to claim about the real contribution of a deep learning model as a feature learning model. Therefore, although our results are lower, our system achieves very promising results without any feature-engineering. The classification of DDIs remains an unsolved challenge in scientific texts, such as MedLine abstracts, and this is primarily because the size of the training dataset is not enough to learn the features, which are more appropriate for the extraction of DDIs from MedLine abstracts. Thus, it is crucial to increase the size of the DDI-MedLine dataset. The same problem occurs with the classification of the *advice* and *int* DDI types, which have very low frequency in the DDI corpus, and therefore, their results were worse than those obtained for the *mechanism* and *effect* types.

Comparing with previous works that did not use deep learning methods, we propose an automatic feature-learning method with 62.23% in *F*_1_ that is a suitable alternative for the classification task without any external information. It should be noted that these systems with higher classification rate (10, 11) used an ensemble of kernel methods with an extensive feature set built from a demanding feature-engineering task. In the related work, we also described recently developed systems for DDI classification based on deep-learning methods, such as RNN or CNN. Unlike previous works, we performed an exhaustive and detailed study of possible settings (in particular the filter size, word and position embeddings) of the CNN architecture, and we performed an in-depth analysis of the results for each type of DDI and over each dataset of the DDI corpus. We plan to study the effect of adding additional layers to this architecture and use the two-step classification (detection and classification of each DDI) as (7). Furthermore, we plan to implement other deep-learning architectures for DDI classification, e.g. recurrent neural network, exploring its parameters without external features as in the present work.

With respect to the CNN hyper-parameters, our experiment results showed that the random initialization of the input word vectors realized a better performance than the pre-trained word embedding models. This may be because these models are learned from text collections such as Wikipedia or MedLine, which do not contain texts which are similar to those on drug package inserts. Most texts of the DDI-DrugBank dataset were obtained from these kinds of documents. In future work, we plan to acquire a wide collection of drug package inserts, and use them to train a word embedding model in order to study the effect of this model on the performance of our proposed system. We also studied the effect of the word2vec parameters, and we can conclude that both architectures (i.e. skip-gram and CBOW) achieved very similar results. However, it is recommended that CBOW be used because it has a significantly less computational complexity compared to skip-gram. For the other word2vec parameters, the default setting used in the C version of word2vec appears to give the best performance. The filter size is another parameter that significantly affects the model performance. Although the early assumptions were that a large filter size would provide better results because biomedical sentences are usually very long, our experiments confirmed that the best filter was (2, 4 and 6). With respect to the effect of position embeddings on the performance, their implementation generally appears to give improved results, being 10 dimensions slightly better than 5.
